# Age-related changes in platysma insertion height and the clinical role of high-resolution ultrasound in the elderly

**DOI:** 10.1016/j.jpra.2025.08.015

**Published:** 2025-08-21

**Authors:** Villiam Vejbrink Kildal, Frank O.F. Reilly, Paata Pruidze, Lukas Reissig, Wolfgang J. Weninger, Chieh-Han John Tzou, Stefan Meng, Andrés Rodriguez-Lorenzo

**Affiliations:** a Department of Surgical Sciences, Plastic and Maxillofacial Surgery, Uppsala University, Uppsala, Sweden; b Department of Plastic and Maxillofacial Surgery, Uppsala University Hospital, Uppsala, Sweden; c Department of Clinical Science and Education, Section of Anesthesiology and Intensive Care, Södersjukhuset, Karolinska Institutet, Stockholm, Sweden; d Department of Anesthesiology and Intensive Care, Södersjukhuset, Stockholm, Sweden; e Division of Anatomy, Medical University of Vienna, Vienna, Austria; f BioImaging Austria (CMI), Vienna, Austria; g Plastic and Reconstructive Surgery, Department of Surgery, Hospital of Divine Savior (Krankenhaus Goettlicher Heiland), Vienna, Austria; h Facial Palsy Center, Tzou Medical, Vienna, Austria; i Radiology, Hanusch Hospital, Vienna, Austria

**Keywords:** Facial paralysis, Platysma, Ultrasound, Ultrasonography, Synkinesis, Hypertrophy, Botulinum toxin, Botox, Injections, Nerve surgery, Sequelae, Platysmectomy

## Abstract

**Background:**

There is no consensus on the exact insertion height of the platysma muscle, and the influence of aging on its cranial position remains unexplored. Additionally, clinicians currently rely solely on clinical examination to assess the muscle—a limitation that may contribute to complications when treating platysma synkinesis with botulinum toxin injections. This study aimed to determine the cranial insertion height of the platysma in an elderly cohort, explore how aging affects its cranial position, and validate high-resolution ultrasound as a non-invasive tool for visualizing and assessing the muscle in the neck and face.

**Methods:**

Thirty-eight hemifaces were studied in body donors of advanced age (mean: 83.5±8.7). Using high-resolution 22 MHz ultrasound, the platysma muscle was assessed in the neck, and the cranial insertion height was established within a coordinate system and verified through anatomical dissection. The effect of age on insertion height was analyzed using linear mixed-effects models.

**Results:**

The cranial platysma insertion height was found 2.9 ± 1.0 cm above the mandibular angle. Increasing age was associated with a decrease in insertion height by 0.54 mm per year (*p*<0.05). Ultrasound successfully identified the platysma in the neck in 34 of the 38 hemifaces but underestimated the cranial insertion height by 0.84 cm (mean).

**Conclusions:**

The platysma muscle often extended into the midface, but increasing age was associated with a reduction in its discernible insertion height. High-resolution 22 MHz ultrasonography of the platysma can reliably assess the platysma and guide treatment in the neck in elderly patients but underestimates the cranial insertion height in the face.

## Background

Facial synkinesis, a common sequela of facial paralysis, arises from aberrant regeneration of injured nerve axons, leading to involuntary muscle contractions in one facial area during voluntary movement in another.^1^ Within the scope of facial synkinesis, platysma synkinesis is a unique facet. Often characterized by noticeable contractions in the neck,^2^^,^^3^ platysma synkinesis can also be a less-recognized contributor to lower lip and midface distortion, as the insertion height of the platysma muscle fibers in the face varies.^4^ The condition significantly impairs facial function, necessitating effective treatment to enhance quality of life.^1^^,^^5^, ^6^, ^7^ Botulinum toxin injections to the neck portion of the platysma is the gold standard treatment for platysma synkinesis.^8^ However, a recent systematic review demonstrated a complication rate of 15.4 %, with 5.2 % of patients experiencing dysphagia and 1.3 % experiencing neck weakness.^9^ These complications likely arise from inaccurate needle placement outside the platysma muscle, resulting in botulinum toxin being deposited in surrounding fascial or connective tissue planes, where it may diffuse to adjacent muscles. While dosage could play a role here, dysphagia also occurs with smaller dosages.^9^ Correct injection technique has been emphasized to prevent diffusion into surrounding muscles and reduce complications,^9^ but the lack of methods to assess the platysma muscle, aside from clinical examination, makes it difficult to verify needle placement within the muscle during injections.

The lack of assessment methods for the platysma has significance not only in the neck, but also for its facial portions. Anatomical data on the insertion height of the platysma in the face remain limited. Historically, anatomy textbooks described the superior border of the platysma as ending at the inferior edge of the mandible, with no midface involvement,^10^, ^11^, ^12^, ^13^ whereas a small number of recent studies suggest that the platysma extends further into the midface.^13^^,^^14^ Clinically, identifying the platysma insertion height could improve the diagnosis of platysma involvement in midface synkinesis and also serve as an aid in platysma myectomy planning by determining the platysma extension pattern in the face, as defined by Bae et al.^14^ However, no standardized methods exist for routine evaluation of the platysma muscle beyond clinical examination. Clinical assessment of the facial portion of the platysma is challenging due to the thin structure of the muscle at its facial insertion, variability in cranial extension patterns, and the overlapping interactions of nearby muscles.^4^^,^^13^, ^14^, ^15^, ^16^ In elderly patients, the frequent occurrence of age-related sarcopenia^4^^,^^16^ adds further complexity. The lack of reliable evaluation techniques presents clinical challenges, where effective diagnosis and treatment require precise assessments, and in research, where progress is hindered by the inability to non-invasively measure muscle characteristics. Developing methods to address these gaps is important for advancing both clinical and academic understanding of the platysma.

High-resolution ultrasound has emerged as a useful bedside tool for perioperative assessments in plastic surgery,^17^, ^18^, ^19^, ^20^, ^21^, ^22^, ^23^, ^24^, ^25^, ^26^ and has recently been introduced for diagnostic and treatment purposes in facial paralysis.^27^, ^28^, ^29^, ^30^, ^31^ The platysma in the neck has been studied using ultrasound previously, but not the superior parts of the muscle in the face.^32^, ^33^, ^34^ This imaging modality could assist in evaluating platysma hyperkinesis by quantifying muscle thickness differences. In the neck, it may guide botulinum toxin administration, optimizing treatment and reducing complications. For midface synkinesis, it could determine insertion height and extension patterns, clarifying the extent of platysma involvement. Establishing the reliability and precision of ultrasound as a tool for evaluating the platysma in the neck and face is essential for its effective clinical application, especially in the elderly where age-related sarcopenia may limit the accuracy of such assessments.^4^^,^^16^ Also, while the effects of aging on the neck portion of the platysma are documented,^35^ the impact of aging on its superior portion remains unexplored, highlighting a gap in understanding platysma anatomy. Given the distinctive thinness of cranial platysma fibers tapering into the face, it is relevant to assess whether ultrasound can reliably identify these fibers in elderly patients.

This study aimed to investigate the cranial insertion height of the platysma muscle in the face, with particular emphasis on age-related changes in its position. We also evaluated the accuracy of high-resolution ultrasound in assessing both the cranial insertion and the muscle in the neck. By examining the impact of aging on muscle position and the reliability of ultrasound, our goal was to improve clinical evaluation and treatment of platysma synkinesis in elderly patients.

## Methods

The study was performed in accordance with local rules and regulations and was approved by the ethical institutional committee in Austria (EC number:1402/2020, with an amendment). Written consent was obtained from all donors throughout their lifetimes.

### Data

Thirty-eight hemifaces from 19 non-embalmed, fresh frozen, body donors were included. The average age was 83.5 ± 8.7 years (range 67–100 years), with 10 female and nine male donors. The most superior insertion point of the platysma muscle in the face was determined and measured relative to a line from the angle of the mandible to the inferolateral corner of the orbit (hereafter called the angulo-orbital line; see Figure 2). First, each hemiface was examined by a radiologist using high-resolution ultrasound before dissection to assess the most superior insertion site of the platysma muscle in the face, with a standard clinical ultrasound system and a high-frequency (22MHz) hockey-stick probe (Aplio i800 and i22LH8, Canon Medical Systems Europe B.V., Zoetermeer, The Netherlands). At the time of ultrasound assessment, the skin on the face, neck, and chest of all the specimens was intact.

### Ultrasound examinations

The length of the angulo-orbital line was measured with a ruler. The neck was then scanned to identify the platysma muscle through both the axial and sagittal ultrasound scanning planes. If detected, the muscle was traced over the mandible into the face until the muscle fibers were no longer visible on the ultrasound screen. The facial portion of the platysma was scanned systematically from posterior to anterior to determine the superior insertion of the muscle. The superior-most part of the platysma muscle, identified using ultrasound, was determined and marked on the skin. The skin marking was then located in a coordinate system, with the angulo-orbital line (described above) making up the y-axis and x-axis as the line perpendicular to the y-axis running through the mandibular angle, approximately along the inferior border of the mandible (Figure 2). Thus, the ultrasound measurements provided two measurements for each hemiface: one along the y-axis and one along the x-axis. The vertical measurements along the y-axis were performed from the mandibular angle. Horizontal measurements along the inferior mandibular border (x-axis) were considered positive if the cranial-most insertion height was found anterior to the angulo-orbital line, and negative if found posterior to it.

### Anatomical examinations

Within 24 h, detailed dissections were carried out by two senior plastic surgeons and an anatomist using loupe magnification, blinded to the ultrasound assessments. Standard surgical instruments were used to remove the skin and adipose tissue down to the platysma muscle layer. When the platysma was fully exposed, the most superior insertion point of the muscle was determined in the face, and two new measurements were taken along the coordinate system for each hemiface (Figure 2).

### Statistical methods

Descriptive statistics were calculated, with data presented as means and standard deviations (mean ± SD). Given the repeated measurements within each patient (two platysma measurements per patient), linear mixed-effects models were employed to assess the impact of age, sex, and facial side on the insertion height, while adjusting for the length of the angulo-orbital line of each patient. To compare dissection and ultrasound measurements, the same model was applied, but with the method (dissection versus ultrasound) as the sole independent variable. The donor ID was incorporated as a random effect. Data normality was visually assessed using histogram plots. A p-value of less than 0.05 was considered statistically significant. Statistical analysis and plotting were conducted using R version 4.4.0 (2024-04-24).

## Results

**Figure 1:**
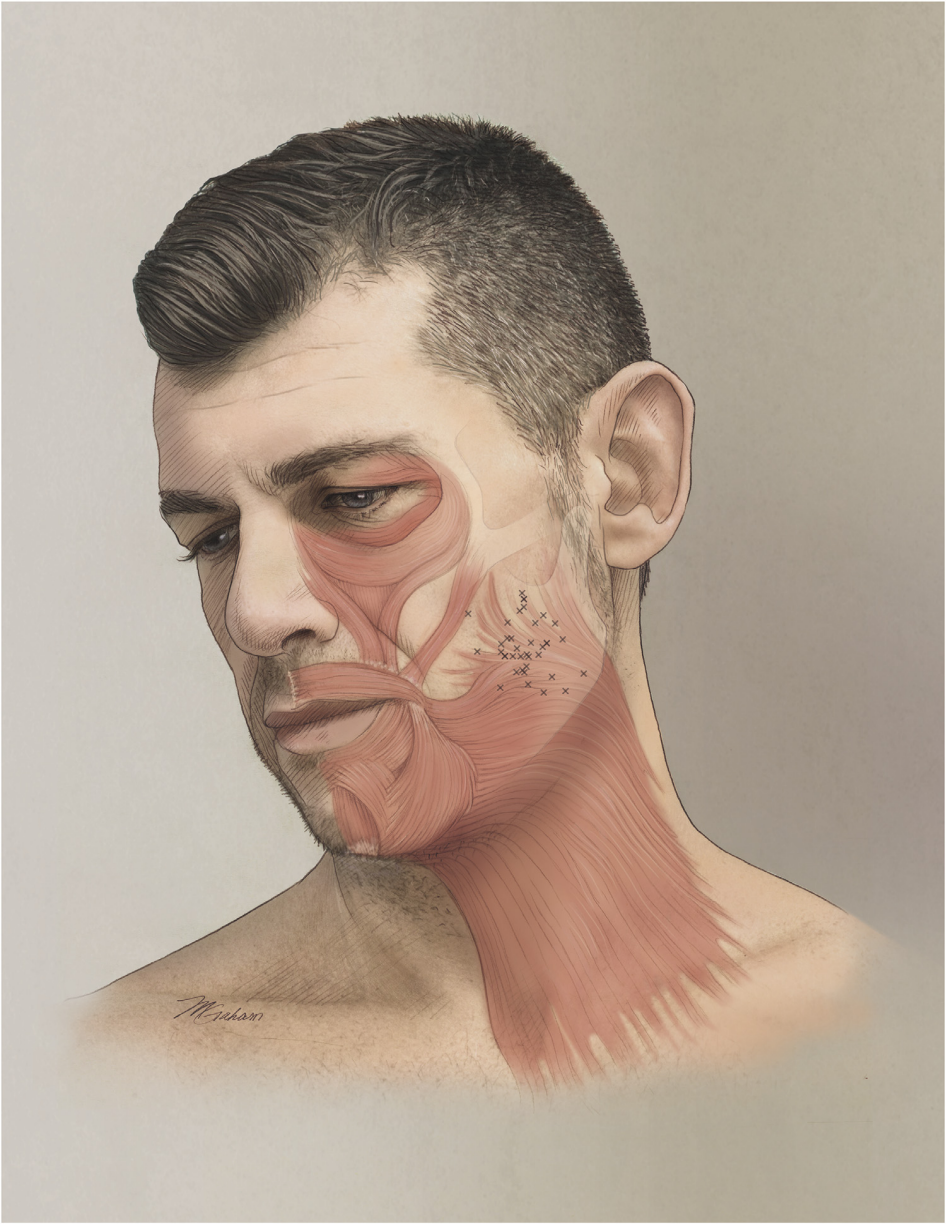
Illustration of the platysma muscle inserting into the midface. The bony landmarks used for measurement are visible (mandibular angle and inferolateral corner of the orbit). The small crosses mark the maximal insertion heights of all individual hemifaces from the dissection measurements.

The line from the angle of the mandible to the inferolateral corner of the orbit (angulo-orbital line, the y-axis) was 9.8 ± 0.9 cm (range 7.8–11.6cm, *n*=38). The dissections located the maximal platysma insertion height at a mean height of 2.9 ± 1.0 cm (range = 0.8–5.2, *n*=38) above the mandibular angle along the angulo-orbital line (the y-axis) (see Figure 1 for a visual representation of all measurements). Horizontally, the maximal insertion height was found at a mean of 1.3 ± 0.8 cm (range = 0–3, *n*=38) anterior to the angulo-orbital line (along the x-axis). Increasing age was associated with a decrease in insertion height of 0.54 mm per year (*p*<0.05), with no significant differences observed based on sex or facial side. A 1 cm increase in the length of the angulo-orbital line was associated with a 0.51 cm increase in insertion height (*p*<0.05).

**Figure 2:**
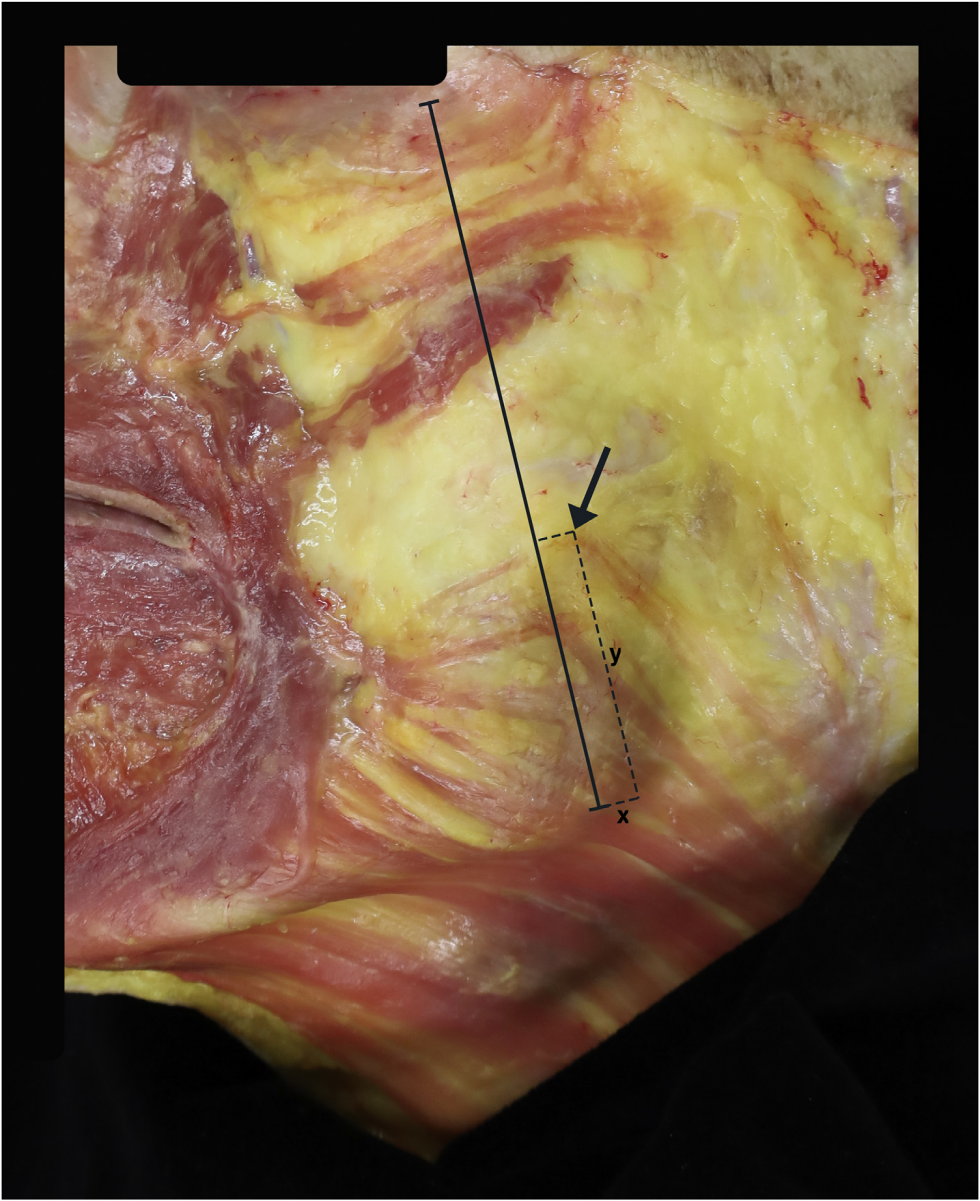
Example image from the dissections. The solid black line marks the distance from the mandibular angle to the inferolateral corner of the orbit (angulo-orbital line). The arrow marks the highest point of platysma muscle fiber insertion detected in this specific hemiface. The “y” marks the maximal insertion height of the platysma muscle along the angulo-orbital line (the y-axis). The “x” marks the horizontal distance from the line to the highest point of insertion along the x-axis (approximately along the inferior mandibular border) (a negative number in this specific hemiface, as the highest platysma insertion point lies dorsal to the line).

**Figure 3:**
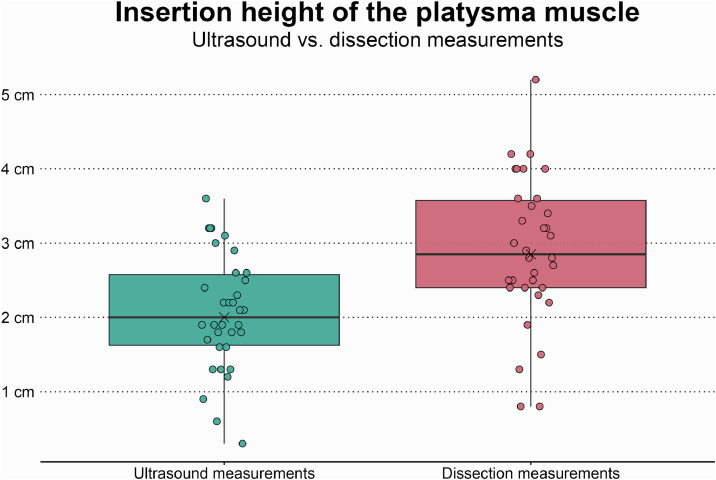
Boxplots comparing the ultrasound and dissection measurements of the maximal platysma insertion height in the face (measured from the mandibular angle along the angulo-orbital line seen in Figure 2). Each point represents a single measurement of a single hemiface. The black crosses represent mean values, and the horizontal black lines represent median values.

**Figure 4:**
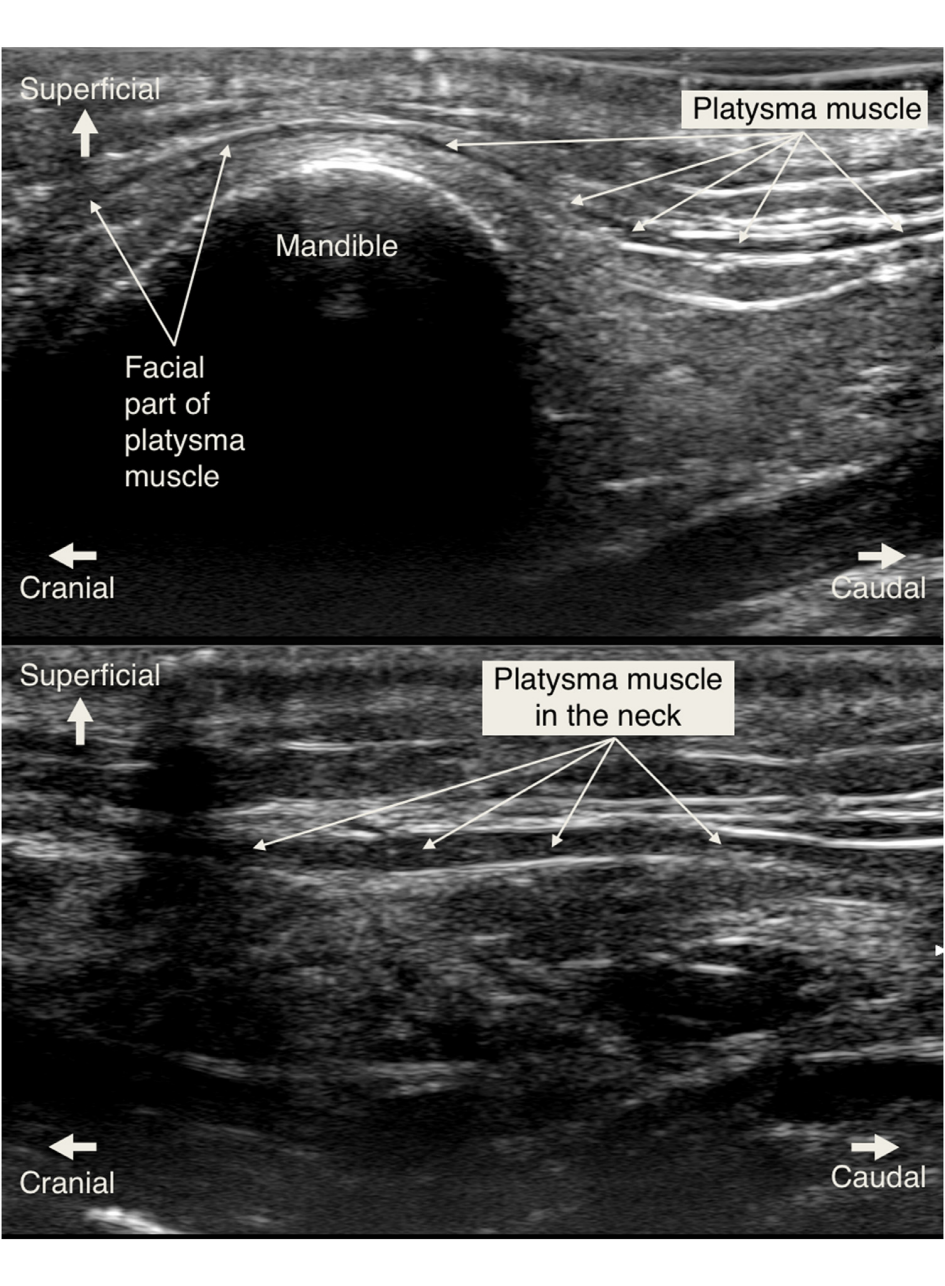
Images showing the sonographic visualization of the platysma muscle. In the face (top image), where it is thinner, and, in the neck (bottom image), where it is thicker and more easily visualized. In the face (top image), the cranial part of the platysma above the mandible is approximately 0.25 mm thick, tapering into the face, while the more caudal part is 0.5 mm thick and lies at a depth of 5 mm. In the neck (bottom image), the platysma muscle is thicker and easier to visualize. Here, the muscle is approximately 1 mm thick, lying at a depth of 5 mm.

Using ultrasound, the platysma muscle in the neck was readily detected and traced in 34 of the 38 hemifaces. In four hemifaces, the platysma was not discernible using ultrasound. The ultrasound assessment located the maximal platysma insertion site at a mean height of 2.1 ± 0.8 cm (range=0.3–3.6, *n*=34) above the mandibular angle along the angulo-orbital line (the y-axis), underestimating the insertion height by 0.84 cm (*p*<0.001) (box plots with comparisons are shown in Figure 3). During dissection, all four platysma muscles that were not detectable by ultrasound were thin and hypoplastic. Examples of ultrasound images of the platysma muscle are presented in Figure 4, where the difference between visualization in the neck and face is demonstrated.

## Discussion

The current study confirms recent findings that the platysma inserts higher in the face than previously believed, while providing novel information regarding the impact of aging on the platysma muscle. Specifically, we found that the muscle inserts at 2.9 cm above the mandibular angle, but that increasing age is associated with a decrease in discernible insertion height, at a rate of 0.54 mm per year. We also demonstrated that high-resolution 22 MHz ultrasound can reliably be used to evaluate the platysma muscle in the neck, thus providing a supplementary tool for assessing and treating platysma muscle synkinesis, even when the platysma is affected by age-related thinning. However, ultrasound underestimates the insertion height in the face of elderly individuals, likely due to age-related platysma sarcopenia.

The dissections revealed that the platysma fibers extended 2.9 cm above the mandibular angle, corroborating the scarce literature showing that the muscle insertion lies more cranial than previously believed.^13^^,^^14^ Numerous studies have explored the anatomy and role of the platysma muscle in facial rejuvenation,^13^^,^^36^, ^37^, ^38^, ^39^, ^40^ but its cranial insertion height has been overlooked in the literature.^13^^,^^14^ Consequently, this is likely an under-diagnosed and under-treated area of facial synkinesis. Recently, a small number of authors have explored the insertion height in the face. During intraoperative measurements in a cohort of presumably younger patients of undisclosed age undergoing rhytidectomies, Shah and Rosenberg found that the platysma extended, on average, 3.98 cm above the inferior border of the mandible.^13^ Similarly, Bae et al., describing platysma extension patterns using cadaveric dissection in a cohort with an average age of 71 years (range 41–93), found that the posterior fibers of the platysma muscle extended a mean of 17.1 mm above the mandibular angle, suggesting that elderly patients have lower insertion heights.^14^ The 2.9 cm measured in this study, in an even older cohort, suggests that the muscle can insert high in the midface even in older individuals. A more cranial insertion of the platysma in the face leads to a greater number of muscle fibers being recruited in the lower and middle parts of the face during synkinesis. Thus, the insertion height of the platysma muscle should affect the severity of facial synkinetic symptoms; however, this has not been proven.

Despite the high cranial insertion, the study also identified a novel association between aging and a significant reduction in the platysma muscle's discernible insertion height, showing an annual decrease of 0.54 mm. These changes, likely driven by age-related sarcopenia and structural remodeling, thus appear to impact the cranial extension of the muscle. Such anatomical alterations may have implications for the diagnosis and management of facial paralysis and synkinesis, particularly in older patients. Further research is required to clarify the mechanisms behind this decline, when it begins, and its clinical significance.

Using 22 MHz high-resolution ultrasound, the platysma muscle in the neck was well-defined and readily visualized and traced in 34 of the 38 hemifaces. Given that the neck portion of the platysma muscle is the primary site for therapeutic intervention, we conclude that high-resolution ultrasound can serve as a reliable and versatile tool for various applications, even in the presence of sarcopenia. Specifically, it can guide botulinum toxin injections and potentially reduce the risk of complications, as demonstrated in the supplemental video showing the notable accuracy of real-time, ultrasound-guided platysma injections. It could be used to evaluate platysma thickness to diagnose and quantify hyperkinesis and hypertrophy, and assess active contraction of the platysma which enables dynamic evaluation of the presence, location, and degree of synkinesis. Regarding the cranial insertion height in the face, high-resolution ultrasound could detect the cranial extent of the platysma muscle fibers, on average, up to 2.1 cm above the mandibular angle. The ultrasound assessment thus underestimated the insertion height of the platysma muscle in elderly patients by 0.84 cm (Figure 3). This underestimation is important to highlight, as it limits the reliability of assessing the cranial portions of the muscle in the face. It must, however, be interpreted within the context of an elderly cohort. Platysma muscle sarcopenia was widespread, a common finding in subjects of advanced age^4^^,^^16^ which complicates adequate sonographic imaging. In the four hemifaces (two cadavers) where the platysma could not be located using ultrasound, dissection revealed thin and hypoplastic muscle fibers, barely distinguishable from the subcutaneous tissue, and the age at the time of death was 100 and 85 years, respectively. In contrast, patients with platysma synkinesis often present with platysma hypertrophy on the affected side,^41^ which can make the muscle directly palpable in the neck. The thicker muscles in patients afflicted with platysma synkinesis, as well as in younger patients without age-induced sarcopenia,^13^ should facilitate ultrasound assessment.

### Limitations

Several limitations must be acknowledged. The studied cohort did not have platysma synkinesis and hypertrophy in life, which likely led to reduced accuracy and a greater underestimation of insertion height compared to a cohort with hypertrophic platysma muscles. On the other hand, the cohort’s composition of typical elderly individuals with age-appropriate platysma muscles strengthens the generalizability of the findings to the general elderly population, emphasizing that ultrasound assessment of platysma insertion height may be less accurate in older adults with sarcopenia. In this study, a 22 MHz ultrasound probe was used. A higher imaging resolution would result in higher visual detail but at the cost of tissue penetration. As the platysma lies less than 1 cm below skin level, a higher probe resolution would likely provide a better balance between resolution and depth, and, thus, more accurate assessments of the thinnest cranial parts of the muscle. Ultrasound measurements were performed by a radiologist to determine achievable outcomes and provide a gold standard. Future studies should determine whether plastic surgeons can achieve a similar degree of accuracy. The anterior parts of the platysma were not studied in detail because the goal of the study was to determine the ability of ultrasound to detect the highest insertion point of the muscle, which was found more posteriorly. Furthermore, the platysma thickness was not quantified. Lastly, ultrasound examinations in living patients can be aided by voluntary muscle activation, which is not possible in cadaveric specimens, potentially making in vivo assessment more accurate than in our anatomical measurements.

## Conclusions

This study confirmed that the platysma muscle inserts more cranially in the face than previously recognized, but also that increasing age was significantly associated with a lower discernible insertion height by 0.54 mm per year. High-resolution ultrasound reliably visualized the platysma muscle in the neck and may be a valuable tool for evaluating and treating platysma synkinesis, even in the presence of age-related thinning. However, cranial insertion height was underestimated, likely due to age-related platysma sarcopenia, and the benefits of using higher-resolution probes warrant further investigation. These findings may have implications for the diagnosis and management of facial synkinesis, particularly in older patients.

## Data availability

Data is available upon reasonable request.

## Financial disclosure statement

This research was financed in part by the Swedish Research Council’s funding for clinical research in medicine (ALF) and by Stenholms stipendium.

## Declaration of competing interest

The authors declare no conflict of interest.

## Appendix Supplementary materials

Supplemental Video Ultrasound-guided, in-plane injection of the platysma muscle. The muscle appears hypoechoic (dark) with hyperechoic (bright) surrounding connective tissue borders. It lies approximately 0.4 cm below the skin surface and measures 0.4 mm in thickness. A 27G needle is introduced from a caudal approach, and approximately 0.2 ml of fluid is injected intramuscularly while the needle dissects along the length of the muscle in a cranial direction. No fluid is deposited outside the muscle. Depth markers are visible on the right side of the image, with “1” indicating a depth of 1 cm.). Image, video 1
